# Realising the European network of biodosimetry: RENEB—status quo

**DOI:** 10.1093/rpd/ncu266

**Published:** 2014-09-09

**Authors:** U. Kulka, L. Ainsbury, M. Atkinson, S. Barnard, R. Smith, J. F. Barquinero, L. Barrios, C. Bassinet, C. Beinke, A. Cucu, F. Darroudi, P. Fattibene, E. Bortolin, S. Della Monaca, O. Gil, E. Gregoire, V. Hadjidekova, S. Haghdoost, V. Hatzi, W. Hempel, R. Herranz, A. Jaworska, C. Lindholm, K. Lumniczky, R. M'kacher, S. Mörtl, A. Montoro, J. Moquet, M. Moreno, M. Noditi, A. Ogbazghi, U. Oestreicher, F. Palitti, G. Pantelias, I. Popescu, M. J. Prieto, S. Roch-Lefevre, U. Roessler, H. Romm, K. Rothkamm, L. Sabatier, N. Sebastià, S. Sommer, G. Terzoudi, A. Testa, H. Thierens, F. Trompier, I. Turai, C. Vandevoorde, P. Vaz, P. Voisin, A. Vral, F. Ugletveit, A. Wieser, C. Woda, A. Wojcik

**Affiliations:** 1 Bundesamt für Strahlenschutz, Salzgitter, Germany; 2 Public Health England, Chilton, UK; 3 Helmholtz Centre Munich, Neuherberg, Germany; 4 Universitat Autonoma de Barcelona, Cerdanyola del Valles, Spain; 5 Institut de Radioprotection et de Sûreté Nucléaire, Fontenay-aux-Roses, France; 6 Bundeswehr Institut für Radiobiologie/Universität Ulm, Ulm, Germany; 7 National Institute of Public Health Romania, Bucharest, Romania; 8 Leiden University Medical Center, Leiden, The Netherlands; 9 Istituto Superiore di Sanità, Rome, Italy; 10 Instituto Superior Técnico, Universidade de Lisboa, Bobadela LRS, Portugal; 11 National Centre of Radiobiology and Radiation Protection, Sofia, Bulgaria; 12 Stockholm University, Stockholm, Sweden; 13 National Centre for Scientific Research Demokritos, Athens, Greece; 14 Commissariat à l’Énergie Atomique, Fontenay-aux-Roses, France; 15 Servicio Madrileño de Salud, Hospital General Universitario Gregorio Marañón, Madrid, Spain; 16 Norwegian Radiation Protection Authority, Osteraas, Norway; 17 Radiation and Nuclear Safety Authority, Research and Environmental Surveillance, Helsinki, Finland; 18 National Research Institute for Radiobiology and Radiohygiene, Budapest, Hungary; 19 Fundación para la Investigation del Hospital Universitario la Fe de la Comunidad Valenciana, Valencia, Spain; 20 University of Tuscia, Viterbo, Italy; 21 Instytut Chemii i Techniki Jadrowej, Warsaw, Poland; 22 Agenzia Nazionale per le Nuove Tecnologie, L'Energia e lo Sviluppo Economico Sostenibile, Rome, Italy; 23 Faculty of Medicine and Health Sciences, Universiteit Gent, Gent, Belgium

## Abstract

Creating a sustainable network in biological and retrospective dosimetry that involves a large number of experienced laboratories throughout the European Union (EU) will significantly improve the accident and emergency response capabilities in case of a large-scale radiological emergency. A well-organised cooperative action involving EU laboratories will offer the best chance for fast and trustworthy dose assessments that are urgently needed in an emergency situation. To this end, the EC supports the establishment of a European network in biological dosimetry (RENEB). The RENEB project started in January 2012 involving cooperation of 23 organisations from 16 European countries. The purpose of RENEB is to increase the biodosimetry capacities in case of large-scale radiological emergency scenarios. The progress of the project since its inception is presented, comprising the consolidation process of the network with its operational platform, intercomparison exercises, training activities, proceedings in quality assurance and horizon scanning for new methods and partners. Additionally, the benefit of the network for the radiation research community as a whole is addressed.

## INTRODUCTION

Following large-scale radiological incidents, a fast medical and radiological triage of patients according to the degree of the radiation exposure will be required. Besides individuals who were actually exposed to high doses of ionising radiation, there will be a large number of distressed people who have not received radiation doses likely to cause acute health effects. A lesson learned from previous incidents is the importance to identify those ‘worried well’ in order to prevent the healthcare infrastructure being overwhelmed and to minimise socio-economic harm. In both contexts, biological and retrospective dosimetry is an essential tool to estimate an actual absorbed dose without being influenced by variations in blood counts or confounding factors. Biological dosimetry will help to identify those individuals, needing extensive medical care due to severe irradiation from people, perhaps with other injuries, but who have not received high doses of ionising radiation^(1)^. Since, in large-scale radiological emergency scenarios, the number of people that may need to be screened could easily exceed the capacity of a single laboratory, biodosimetry networking has been recognised as a pragmatic and important emergency response strategy in several regions of the world^(2)^. A network of six laboratories has been set up, under the patronage of IAEA, covering the whole of Latin America. The US Government is promoting a similar initiative in the USA. A global approach was started by WHO with BioDoseNet^(3)^. National-level networks have been established in Japan^(4)^ and Canada^(5)^. Now, based on the outcome of the TENEB survey^(6)^, a European Network of biological and retrospective dosimetry (RENEB) was initiated^(7, 8)^. It started in January 2012 with a total of 23 organisations from 16 European Union (EU) countries.

## STATUS QUO OF RENEB

### Key aspects

RENEB focuses on five key demands, which are fundamental for a sustainable network^(7, 8)^:

- to create an operational basis for the network based on coordination of existing reliable and proven techniques in biological dosimetry (*operational basis*),
- to ensure that the network remains up-to-date by providing the basis for implementation of appropriate new biological and individualised methods and by expanding through integration of new partners (*development of the network*),
- to assure high-quality standards for reliable dose assessment by implementing a framework for education and training activities, intercomparisons and quality assessment and management procedures (*quality assurance*, *education* and *training*),
- to develop an operational infrastructure within the network and outward, a financial stability based on a long-term funding strategy and to implement an official framework to transform the RENEB project into a legal organisation (*long-term sustainability*),
- to achieve visibility, accountability and sustainability of the established network within the European and global emergency preparedness system and international research community through dissemination of knowledge and a strong linkage of the network to national regulatory authorities, international bodies and platforms (*dissemination activities*).

### Operational basis of RENEB

Currently, the best methods of biological dosimetry are based on the analysis of cytogenetic damage (dicentric chromosomes, micronuclei) in peripheral blood lymphocytes^(9–12)^ and electron paramagnetic resonance in bone and tooth enamel^(13)^.

Additionally to these methods, a number of further developed biodosimetric methods have been introduced, such as premature chromosome condensation (PCC), fluorescence *in situ* hybridisation (FISH) and *γ*-H2AX *foci*^(9, 11)^. In addition, the EPR/OSL method on portable electronic devices, chip cards, although strictly speaking not a biodosimetric method, has been shown to have the potential to be an excellent supplementary individual dosimetry tool^(13)^. Some of these methods are established in several European laboratories^(9)^, but formal networking was lacking, which would facilitate the standardisation of the assays. RENEB now has started to provide such a framework for regular intercomparison studies and accident exercises. Exercises and intercomparisons have been performed comprising the dicentric-, FISH, micronucleus-, PCC-, γ-H2AX- and EPR/OSL-methods. Except for the EPR/OSL techniques, the intercomparisons comprised two parts: (1) analysis of electronically stored images and (2) comprehensive intercomparisons. The latter included shipment, culturing of blood samples, preparation of slides, finding of the target, image processing and finally scoring and dose estimation. With this approach, valuable information concerning the need for harmonisation of the network was obtained.

Although the overall performance of the network partner laboratories was satisfactory, some room for improvement was identified for each technique and was addressed in training events (see ‘Quality Assurance, Education and Training’).

A 2nd comparison is planned for autumn 2014. This exercise will be open to potential new members who showed interest to join the network, as well as to other national and global networks.

### Development of the network

The established network is not designed to be a static or closed consortium; the sustainability will depend on openness and the ability to react in a flexible way towards new situations. Thus, it is a major goal of RENEB to actively identify promising techniques and potential new partners. In order to develop tools for the identification of new technological developments, a multistep strategy was prepared and a similar approach to identify, attract and integrate new network partners was defined. Reporting sheets for new members and new technologies can be found on the RENEB website (www.reneb.eu). The campaign to scout for new partners and techniques was already effective with five laboratories showing an interest to actively participate once the network is established. Also two new technologies were directly reported to RENEB, one of them an amelioration of existing techniques and one a high-throughput system.

### Quality assurance, education and training

The true value of biological dosimetry lies in the speed of classification of persons according to the degree of exposure. In large-scale emergencies, the response time of the network depends chiefly on the efficiency of all labs involved in the response, not only individually but also in coordination. The best operational conditions result directly from the preparedness of the network already before the event; therefore, the requirements include harmonization of procedures, retention of qualified staff, knowledge of the laboratory capacity in crisis situations and common training. As a first step, a survey focussing on the acceptance and demand for training activities of the RENEB partners was performed and an on-line training in image scoring was completed (see ‘Operational Basis of RENEB’). Some need for training was identified by the 1st intercomparison, leading to selective actions and exchange visits of scientists and technicians for some practical training in partner laboratories.

In addition to practical training exercises, seminars on statistics for biological assays and quality assurance were performed^(14, 15)^. A second QA&QM seminar on ISO documents will take place in the autumn of 2014. This training will be based upon the recommendations of the appropriate international (ISO) standards and will establish periodic intra- (for the qualification of individual laboratory staff) and inter-comparisons (for the qualification of the network)^(16)^. The programme will also include theoretical calculations and experimental design. Additionally, informal contacts were installed with European training structures/programmes that have a strong impact in this field. In order to develop long-term QA&QM programme, actions to sensitisation for a QA&QC programme were initiated.

### Long-term sustainability

A meeting on establishing an operational communication structure was held in the autumn of 2013, and a first structure was defined, including links to national and international health care units. Arrangements aiming at long-term sustainability were identified, and as a first step, the joint research interests within the network partners and outside the network were identified through a questionnaire. It became obvious that beyond the use for emergencies, the network with its capability to jointly analyse large numbers of samples is able to significantly contribute to a wider field of radiation research topics. Good examples are long-term follow-up studies on low-dose effects, on individual radiation sensitivity or molecular epidemiological studies. In this context, RENEB will apply to the infrastructure session at the 5th MELODI workshop in October 2014 that will have a major influence on the European Research Area. Links to European radiation key platforms as MELODI^(17)^, EURADOS^(18)^ and NERIS^(19)^ have been initiated. Outside Europe, links to international organisations (mainly the IAEA and WHO) dealing with radiation emergency preparedness and Education & Training platforms (such as those supported by ENEN, ENETRAP, ENSTTI and IAEA) have started.

### Dissemination activities

It is crucial for RENEB to maintain strong links and cooperation with European and international organisations involved in emergency preparedness and response. A promising basis is the already-existing involvement of several RENEB partners in international activities like the WHO BioDoseNet^(3)^ and REMPAN^(20)^ and the IAEA RANET^(21)^. An open web page is maintained (www.reneb.eu), and a secure internal web page with access only for RENEB members is under development. Bulletins (newsletters) with the information about the RENEB project were published in August 2012 and September 2013 and are distributed at meetings and conferences. Information about RENEB and its further development was also provided by poster and oral presentations at relevant radiation research and emergency preparedness meetings. These include meetings organised by IAEA and WHO, as well as by the International Radiation Protection Association, the European Radiation Research Society, the North Atlantic Treaty Organization and Research and Technology Organization (NATO, R&T), the Bundeswehr Medical Academy (CONRAD), the MELODI association and at EPRBioDose. To guarantee a smooth flow of action in an emergency on national level, contacts to the relevant national bodies responsible for biodosimetry arrangements will be further facilitated by national representatives from the RENEB consortium countries.

## FUNDING

This work is supported by the EU within the 7th Framework Programme (grant agreement No. 295513).
